# Henry De Mondeville, the Man and His Writings

**Published:** 1903-04

**Authors:** Charles Greene Cumston

**Affiliations:** Boston, Mass., 871 Beacon Street.


### Henry de Mondeville, the Man and His Writings.

### With Translation of Several Chapters of his Works.

By CHARLES GREENE CUMSTON, M. D., Boston, Mass.

(*Concluded from March Number.*)

Aside from the rules just mentioned one must also have recourse to other rules, which have been treated above in the first chapter, Tractus 2, on the Treatment of Ulcers, and he must select those which he will there find that are necessary for the art he is practising. From the definition mentioned with its explanations, from the divisions given, from the causes and reasons, from the above-mentioned general rules, besides some other things which refer to the above-mentioned matter, the educated surgeon can be sure to see almost the entire modus of how one should operate in general and in accordance with the rules of the art. This modus consists of four parts—namely, (*a*) the general evacuations or purgations; (*b*) of the special and diverting; (*c*) of suitable diet and conduct; (*d*) of local remedies and manual operations, according to the rules of art.

In order to render the fourth point clear five conditions must be noted: (*a*) it is to be remembered that each abscess from which a patient is cured has four stages—namely, the commencement, the development, the height, and the decline. The commencement of an abscess is taking place when some matter begins to distend and thicken a limb, and when positive disturbances of the natural functions of the diseased limb are first noticed, and when nature up to that time has not taken the matter of the abscess under her care. Growth exists from the beginning until the abscess has developed and ceases to do so, and as long as the force is weakened, and while the matter increases; then nature occupies herself with the abscess, but as yet does not successfully combat it. The height of an abscess I call that condition when the lesion neither increases nor decreases in size and when the natural force controls the diseased matter in some way or other. The period of decline is when the abscess commences to decrease and when the accompanying symptoms become milder until the lesion is healed or becomes transformed into some other affection, such as a fistula or an ulcer.

(*b*) It is to be remarked that any of these stages may remain latent for a certain lapse of time; thus the stage which we call the beginning of the abscess may represent the beginning, middle or end of the process, for which reason an abscess may appear to be small for a long time. A similar statement may be made regarding the stage of growth—namely, that occasionally mucous abscess, botia, and tubercular glands and so on, grow continuously for several years, occasionally during the entire life of a patient, and thus the stage of increase has several periods. The same holds good for the height and the decline. If, therefore, in the beginning of an acute abscess, according to writers, local measures, dry, cold and styptica, should be applied, and if these do not cause improvement, and if since they do not counteract, the abscess grows, it is necessary to employ some dissolving remedies in conjunction. From this it follows that the nearer the stage of commencement is to the period of growth the less local cold, dry and styptical remedies are to be applied; on the other hand, the more advanced stage of growth, and the nearer it is to the period of the commencement of the abscess, dissolvents should be added in smaller quantity than repercussiva, and the further away it is from the stage of commencement, stronger dissolvents must be added in preference to the repercussiva.

The same holds good for the nearness or the distance of the height of the lesion relative to the stage of growth, and of the period of decline as regards that of the period of height, and thus the respective remedies must be added and, as the case may require, be added or reduced in amount.

(*c*) It is to be noted that the distinction above mentioned of the stages of abscess and the latent condition of any one of these stages must be considered if the surgeon is to proceed according to the rules and in a minute way. But it will happen occasionally that the process heals although the surgeon is heedless of what we have said, in which case the healing is usually neither good nor prompt and the result thus obtained is more to be attributed as an accident than to the skill of the surgeon.

(*d*) It is to be noted that the modus referred to for the regular healing of an abscess only applies to those lesions which do not suppurate, because suppuration does not belong to the four principal intentions of cure as will be seen from the above-mentioned rules. And supposing that the treatment just mentioned would not suffice to cure an abscess, and that for some reason or some error, suppuration should supervene, the abscess must be ripened as the case may require, and as will be shown later on when describing special kinds of treatment. A ripe abscess is to be opened, observing the rules and cautions enumerated in the chapter on the treatment of ulcers. When it is opened it should be cleansed, after which it must be dried out and the flesh to be renewed, and is to become firm after its removal, all of which is to be accomplished as laid down in Chapter I., on The General Treatment of Wounds, section 7, which discusses the treatment of acute abscess, and the bad condition of those suffering from wounds, as will be shown later when treating the special methods of the treatment of abscess.

The local remedies have, nevertheless, been treated in the present chapter, and also what is going to be mentioned in a separate chapter of the special methods of treatment, and will be found in the Antidotarium, to which the reader is referred.

(*e*) It is to be remembered that beside the professional treatment described, there is still another which is partly based on logical consideration, partly on experience, and which method has a wonderful influence on all abscesses, admitting that they have not as yet arrived at the point of suppuration, and which perchance, the ancients did not know, or which they did not desire to write on, because of its usefulness and easy application. When we are in doubt as to whether an ulcer will or will not suppurate, we should apply the following prescription at once both day and night. It consists of a plaster of marshmallow leaves with their stalks but without the hard stems, and rolled in oakum, moistened with water, boiled under ashes, and then carefully ground. Then from three to fifteen leeches should be applied, their size being selected according to the size of the abscess, the age and the strength of the patient, at one point one after the other, where nature appears to collect the largest amount of pus, and then this point is poulticed by means of hot water and is carefully dried. Then leaves of the leek which have been pounded or ground in warm olive oil, are spread over the abscess and a large area of the surrounding parts, and then a goodly amount of hemp-oakum should be spread over this in order to retain the heat. This is continued for three days with only one change; on the fourth day the plaster, as described above, is again applied and in the morning the leeches, and then the plaster of leek leaves is applied as before. Thus, the abscess with the treatment continuing alternately till the tenth day becomes ripened, is reduced considerably in size, and in most instances will give exit to the pus without resorting to the knife or medicine.

And if an abscess appears ripe and does not open by itself, it should then be incised, observing the necessary rules. When the opening has been made a strip of lard is inserted, because that prevents the opening from closing up, renders the borders of the wound pliable, and thins the pus; thereupon an infusion of wheat flour should be applied with which water and a little oil is boiled to produce the consistency of dough, whereupon the said infusion is daily renewed with the application of the lard on top until a cure is effected.

*Palliative Treatment.*—As to its manner, usefulness and the question as to how many physicians quote it in their works, it may be said that it is similar to what is described in the preface to Oct. II., Tract II. Palliative treatment is to be recommended in three cases: (*a*) when the abscess is simply incurable, such as hidden cancer, or an abscess forms a tumor which arises in the nipple or the eye; (*b*) if the patient is desirous of being freed from the suffering of an abscess; (*c*) if the treatment of an abscess, for example old hemorrhoids, would necessarily be followed by a disease or some worse conditions, such as lepra, hydrops, or mania. The last rules regarding this manner of treatment will be described in a separate chapter.

Regarding the explanation of what has been said, and the subjects relative to them, eleven items are to be observed: (*a*) that abscess, tumor, protrusion or elevation, a thickening, or an unnatural swelling are one and the same thing, in other words, they are synonymical expressions. All that belongs to the species abscess, has many variations, such as exitura, pustula, and the like; (*b*) exitura is different from abscess and the rest, of which each can be applied to any unnatural tumor, whether it produces or must produce suppuration, or be it that such is not the case. But of exitura one only uses it with respect to acute abscess, or one that has accidentally become acute, after suppuration has arisen in it and not before, as will be afterwards mentioned in the chapter referred to. If you observe very marked pulsation or a lasting hardness or warmth, the abscess is then on the point of becoming an exitura. Until this it was nothing of this kind before the mentioned symptoms appear, and this is no abscess; (*c*) it is to be noted that bother and pustula are two different things, because according to Avicenna, bother is a small protrusion or abscess, the entire collection of pus of which is situated outside the flesh, that is to say between the flesh and the skin, and is non-poisonous, while a pustula consists of poisonous matter which eats itself up; (*d*) what Avicenna says in Chapter V. on Complicated Diseases, should be noted: One must not assume that a walled abscess arises from the blood and bile, but is rather one which originates from some matter or other, whether this be warm by its own nature, or be it that warmth has been added to it because of an intervening decomposition, and it is quite permissible to divide these symptoms according to the division of the kind of every matter—namely, phlegmatic, melancholic, and the like; (*e*) it is to be noted that the expressions causa primitiva extrinseca and exterior antecedens, also intrinseca and interior, congestio and congregatio, derivatio and deligatio are identical; (*f*) this definition of the conceptions and terms is most important. Thus, Galen in the Fifth Book on Simple Remedies says: "An error in the things designated by name injures the patient very much . . . ," and he adds "physicians confound and spoil names, and not only the names themselves but also the knowledge of the names and things; (*g*) it should be noted, according to Avicenna in the chapter referred to that abscess is a composite disease, that is, in an abscess one has various kinds of diseases—namely, (1) an unfavorable diathesis, because there will be no abscess unless the humors are defective; (2) defective form, because no abscess will arise without an injury to the outer appearance or without a swelling, a lesion of continuity, since the pus of abscess is imbibed between the parts of the diseased limb and so severs the continuity of the parts; (*h*) it is to be remembered that an abscess is a disease which effects equal parts, equal functions or the entire body. It is a morbus consimilis, because it occurs in similar parts—namely, bones, nerves and muscles; officialis, because it effects limbs of equal functional importance, such as the hand and foot; communis, because it can occur in all members excepting the heart, which on acccount of its importance cannot undergo abscess formation; (*i*) according to Avicenna, in the chapter referred to, the bones are affected by a disease which resembles abscess and the like, because all that takes nourishment will also take a superabundance of nutritive material, such are the bones; superabundance of nourishment, however, is not abscess, etc.; (*j*) Galen in his Tegni places the manner of origin of abscess as follows:

To any one part humors flow, or what comes to the same, they remain in it for its proper nutrition, the member becomes tense, its veins, even the very smallest, are gorged, so that matter which did not previously exist there is present, as may be seen in a tumor of the conjunctiva of the eye; afterwards they leave the veins and enter into the hollow spaces of the parts and are left there by nature, etc.

The manner of origin of pus is described by Galen in his fifth book on Internal Diseases, and in the commentary to the second part of the Aphorisms on the Origin of Pus:

That which is pus or poison, or putrid matter, dirt, scabs, crusts, and the relations of each other and from which matter, which is their effect and in the manner which each is created, all that has been said in the chapter on the Treatment of Ulcers and in the explanations, is to be noted that the disease can be called communis for three reasons: (1) because it attacks all members at the same time at once, as fever does, for instance; (2) because it comprises every kind of disease, inasmuch as it affects similar and general tissues of equal functional importance, and includes to the same extent abscess, scabies, pruritus, serpigo, and similar affections; (3) because it can originate in single organs, such as wounds and ulcers.

Such is the literal English version of the chapter on Abscess and its Treatment, although I must offer an apology for the very rough translation that has been given.

De Mondeville's remarks on the prognosis of wounds are most interesting in many respects, and show the progress made in surgery at the time of his writing. He says that according to the views of ancient writers, a wound whatever may be its character was considered as either absolutely fatal, usually fatal, or not fatal, and that according to Theodoric a wound is either absolutely fatal, not absolutely fatal, or not at all fatal. He then goes on to say that those wounds which the ancient physicians considered as usually fatal, and those considered not absolutely deadly, or not fatal by Theodoric, he considered as not fatal, or at least less dangerous than did his predecessors. He also says that he, and perhaps Theodoric also, considers no injury as absolutely fatal, excepting one which kills the patient before he has been able to take any food, provided that no mistake in the treatment has been made, and he states that if the wounded patient has survived an hour and has partaken of some nourishment, his wound will not kill him unless some mistake in the care of the patient is made.

He points out that all wounds of considerable size, and wherever situated, are usually fatal if they are looked upon and treated according to the methods of the older surgeons, and that it is a peculiar fact that he learned by many years of experience in Paris that flesh wounds of the head, without injury to the bone, are usually fatal, perhaps more frequently than when fracture of the skull occurred at the same time. He explains this by the fact that when the skull is injured, a considerable amount of brain substance becomes evaporated, thus relieving the brain itself in many respects.

That de Mondeville was a sagacious clinician, is shown by his remarks on the general condition of patients, for he points out that some people have a great power of resistance and shows that some quite dangerous wounds will be recovered from in a robust habit, which otherwise would end fatally in a weakly constitution. Wounds of the heart, no matter what their size may be, are always fatal, because this is the most sensitive organ and its normal action is a fundamental condition for the existence of life; and whent his organ is wounded the spiritus and heat and the humors mingle together and produce an ulcer. An insignificant wound becomes most important when seated in a vital organ. If, therefore, a wound reaches the interior of the heart the patient will immediately die, while if the wound be only superficial the patient may live one day but will surely die in the end, because the accumulation of the humors will give rise to an inflammatory ulcer invariably resulting in death.

Injuries to the bloodvessels which extend as far as the digestive tract or the organs of respiration are also absolutely fatal because the blood will escape from the inside of the body through the mouth and the hemorrhage cannot be checked. This is likewise the case of wounds to the trachea which extend as far as the cartilaginous part of the organ, because this latter tissue is dry and on account of its great hardness and dryness union cannot take place. Wounds of the esophagus, pericardium, the stomach, gall bladder, the intestines, especially the jejunum, the uterus, the fundus of the bladder, are fatal if they extend into the interior of these organs, for the reason that they are very rich in nerves and are dry, so that union cannot take place. He also points out that the pericardium and diaphragm are in constant motion, while the intestines and esophagus are subjected to a constant irritation, producing movement, thus preventing healing from taking place.

As absolutely fatal he also considered vast traumatisms, such as amputation of the leg, or crushing of a limb, especially the thigh, and he attributes the mortality in these cases to the vital strength not being sufficient to permit of the healing of such large wounds; but if the strength were sufficient the patient would recover, provided that no important organ were injured at the same time.

De Mondeville says that superficial injuries of vital organs, excepting the heart, no matter how large or severe they may be, are not necessarily fatal if they are treated according to the rules laid down by Theodoric, the reason being that the vital strength is preserved until healing has taken place.

Speaking of wounds of the brain, which appear to be particularly fatal, Theodoric says that he saw a man recover who had lost about a third of the posterior part of the brain, and the patient who was a wood carver lost none of his artistic qualities. De Mondeville says that he has many times removed shot from the brain, and that all the patients recovered. De Mondeville advocated dry dressings in most cases when the wound was fresh, but when he had to deal with one which was already suppurating he first purified it by means of certain remedies, and he points out that no healing can take place until the production of pus has ceased.

After the wound had been rendered clean he applied stimulating and healing remedies.

The first chapter of the third treatise, which treats of incisions, was written before 1314. The remainder of the third treatise was composed after 1316, which was the date of the death of Louis le Hutin, mentioned by De Mondeville. It took our author three years to compose this third treatise which has remained unfinished, and consequently would make the date from 1319 to 1320. Fearing that he would not be able to finish his work, de Mondeville did not complete the third treatise, but composed the fifth, the Antidotarium, because he considered the latter more important.

The complete or fragmentary manuscripts of de Mondeville's surgery number in all eighteen, but to these manuscripts still one more must be added which today is found at the Laurentian Library, at Florence, and contains the provencal translation of de Mondeville's abridged anatomy, reproducing the course of lectures which he delivered first at Montpellier in 1304, and I here append the introduction of this provencal translation as given by Dr. Bos.

*Comensa lo prologue de la notomia de la Surgia de Anric de Mondavilla.*

Al comensamen d'aquesta obra, que es tracha de lati en romans dea la Surgia de Anric de Mondiviala, deves premieyramens saber que es subjet en tota surgia . . .

Lo premier es de la notomia coma defendemen de Surgia.

Lo segon es de la cura universal de plaguas et de concutios.
Lo ters es de curas de totas malautias que non son pas plaguas ni ulceracios ni malautias de foras las quals venon comunamen a totz los membres del cap entro als pes.

Lo quart es de la cura de la strenquaduras et delogaduras et cossemens et plagamens.

Lo quint es l'antitotari.

Et a me sembla que Vicenna es pus savi en la notomia; et Tederic en la cura de las plaguas et Alofranc en la cura de las ulceracios et de las autras malautias procezen trop ben davant tot autres surgies.

Lo premier tractat d'aquest libre que tracta de la notomia est devisat en XII. capitols principalmen et per orde.

This small number of complete or fragmentary manuscripts, nineteen in all, of a manual composed for students and practitioners, appears to show that the work of de Mondeville did not have any great success. It was nevertheless a good manual, giving the status of the knowledge of his time, but it was incomplete and had as a competitor the almost contemporary work of Lanfranc, whose teachings had had perhaps a greater vogue and whose treatise on surgery had already been completed.

Like all works which are put aside as soon as science has made some little progress, the treatise on surgery by de Mondeville had only a short existence, and the work by Gui de Chauliac caused the name of de Mondeville to be completely forgotten, but to him will ever be attached the honor of having been the first French surgeon who wrote a book on surgery. But this was not his only merit, as I shall endeavor to point out in what is about to follow.

There is every reason to believe that de Mondeville was instrumental in founding the College of Surgeons, which truly was the work of Pitard, and he was obliged to use his influence with the King of France, and with Prince Charles de Valois, in order to complete this useful work.

It was at about the time when he returned to Paris in 1306, that de Mondeville put into execution the project that he had conceived some time past, to write a complete treatise on surgery for his students. He was a persevering worker and most intelligent, and he had read and meditated on all the works of the most celebrated surgeons of his day, the names of whom I have already given. He had seen the practice of several of these noted men either at Montpellier or at Paris, and under the directions of his master, Pitard, he became familiar with all the operations.

De Mondeville had already taught with great success, and had a profound knowledge of the ancient sciences and the best Arabian writers, and while in Italy he had heard the lectures of the most renowned surgeons.

Nothing in his education was wanting, and besides talent he had an independent turn of mind, exempt from all cupidity and envy, contenting himself with simply the necessaries of life, and being a bachelor and consequently not obliged to look after the necessities of a family, he found that he was not even able to carry out the editing of his surgical treatise. He believed that his work would be all the more opportune because he found, as he says, that the surgeons of his day were disposed to serious study, and he was in hope that his treatise might be conducive to their desire for learning.

De Mondeville's life was well filled. He taught surgery and anatomy in Paris before a large assemblage of students and persons of distinction, and as I have already said he was the surgeon to the king and to the army. But the occupations and his deplorable condition of health prevented him from fulfilling the vast program that he had laid out for himself. In a few years he completed the first two treatises of his work which he read in 1312, "Publice, absque collecta, cum scholorium medicianae et aliorum aliquorum intelligentium maxima et nobilissima comitiva civium, curialium et pertranseuntium advenarum." But he soon received an order to follow the army commanded by the king's brother, Charles de Valois, which was to go to Arras and to England, and he was obliged to bid good-bye to his students and his much cherished studies. However, at his entreaty the king allowed him to return to Paris before the end of the expedition, and he was thus enabled to again take up the work which was the principal charm of his life.

He commenced the third treatise of his work and finished the first two sections, when at this point he was again obliged to stop on account of his bad health. In a melancholy state of mind he wrote the following touching words regarding his condition:

Non cyrurgicus se exaltet, sed timens Deum qui amicus sapiens est, confidat de ipsius maxima largitate et suae plenituvine potestatis, sub quibus, quasi miraculose et de gratia speciali, eanguidus vivo et jam vixi continue per duos annos contra commune judicium medicorum. Rogans, insuper et supplicans Creatorem, ut sicut ipse Ezechiae regi vivendi spatium prolongavit, ita et vitam mihi prolunget, si placet, ad profectum commune, donec deintaxat, possim perficere opus peresens ut ad ejus complementeum concrescat ut pluvia, doctrina mea, et fluat ut ros, eloquium meum.

He hastened to trace in outline the titles of the chapters comprising the third part or doctrine of the third treatise that he intended to compose, and to outline the fourth which was given up to the study of fractures and dislocations. And, lastly, as I have already said he began to edit his Antidotarium. Although both phthisical and asthmatic he was able to finish this important therapeutical treatise. Such, in a few words, are the principal points in the life of this excellent man.

The first treatise, as I have already said, is given up to the study of anatomy, particularly that which applies to the practice of surgery and what would at the present day be termed regional anatomy. De Mondeville says that it is quite sufficient for him to treat briefly grosso modo the anatomy of the human body, nec intendendo ipsam radicitus nec ad unguem, but to consider it in its relation to surgery. It is very doubtful whether or not de Mondeville had dissected, because in his day this practise was extremely rare. But the illustrious surgeon of the King of France says that he first had had the idea of joining to his anatomical descriptions a certain number of drawings which would speak to the eyes, and thus impress more deeply in the memory the elements of a science which is so indispensable both to physicians and surgeons. It was, however, necessary to wait the advent of that great genius, Vesalius, aided by the artists of the Rennaissance, to see the great surgeon's idea bear fruit. The drawings that de Mondeville has left us are extremely small and of only slight value, and it is probable that for the illustrations of his lectures he must certainly have had more correct and more expressive plates.

The second treatise of de Mondeville's works is consecrated to the study of wounds, contusions and ulcers, and was the object of his particular attention. It is preceded by a preface and long prolegomenon under the title of, Notabilia introductaria ad totam cyrurgiam.

The following lines regarding required qualities of a surgeon are in truth remarkable. He says:

> The surgeon if he is desirous of operating well, should in the first place frequent those places where talented surgeons often operate; he should apply his entire attention to their manner of acting and fix it in his memory, then to exercise himself by operating under the guidance of his venerable masters. The surgeon must be endowed with a natural genius, because it is dangerous to operate when only guided by what is written in books, without consulting one's own proper inspiration and a sound judgment. Ingenium naturale adjuvat artem et naturam regentem. Necessarium est cyrurgicum fulgere ingenio naturali . . . He is not a good surgeon who does not know the art and science of medicine and especially anatomy . . . The surgeon should be fairly audacious; should not discuss questions before the laity; he should operate with prudence and sagacity; he should never commence perilous operations unless he has provided everything in order to avoid danger; he should have a well made hand, the fingers long and slender, supple and sure. He should promise health to all his patients; he should not conceal from the parents or the friends the dangers that may arise; he should avoid as much as possible difficult cures; he should never undertake hopeless cases; he should give the poor gratuitous care; if possible the rich should pay well; he should not sing his own praises; he should not cover his colleagues with blame; he should not cause envy among them; he should work always with the idea of acquiring a reputation of probity; he should be reassuring to his patients by kind words and acquiesce to their requests when nothing harmful will result from them as to their cure. . . . From all this it follows that the perfect surgeon is even more than the perfect physician, and that the former has need of a condition which the second may not possess—namely, manual dexterity.

His introduction to surgery is less a discourse than a series of twenty-six articles classed under the head of Notabilia. Under this heading our author considers a large number of points of great interest for the history of the art and practice of surgery in the middle ages, such as the conditions necessary for becoming a good surgeon, remarks on quackery and empiricism; the relationship between physicians and surgeons and a large number of other questions relating to medical ethics.

It is well known what enmity there was between physicians and surgeons at a later period in the history of medicine, and dating from this time the fight was continued, and many were the debates which had for object to decide what diseases should be treated by the surgeon and what by the physician. It was finally decided that wounds, abscesses, ulcers, hemorrhoids, various skin diseases, all external affections of the head, arms and legs, the seat of which could be discovered, although external appearances were absent, such as pains of the joints and hands, deafness, decreased vision, etc., should belong to the domain of surgery and should only be treated by surgeons.

As to internal affections of the head and those arising in the thorax or abdomen, excepting perhaps dropsy and a few other similar affections, it was decided that they should be treated by physicians only.

Henri de Mondeville was opposed to the brutal separation of surgery from medicine, as well as against quackery and the pretensions of the clergy, and all abuses which at this epoch dishonored science.

His treatise on wounds is without doubt the most important and interesting part of his writings.

Immediate ligature, which was indefinitely indicated by Celsus and Galen, will be found perfectly described by de Mondeville, who in no way claims the idea as his own, but gives all the honor to Lanfranc. This method of ligating was usually employed when other means of hemostasis had failed. The skin was incised so as to expose the wounded artery, which was then drawn out with a hook or pincers, and then according to the text the vessel was twisted. It was then tied and the borders of the wound were brought together by sutures, allowing the ends of the ligature to be brought out. The ligature was removed as soon as granulation tissue had developed to an advanced degree. It was only reserved for Ambroise Paré to apply the ligature of arteries in amputations. De Mondeville expressed badly founded ideas of the success of this method, because he was unacquainted with the phenomena of the spontaneous dropping off of a ligature, and he says:

Either the ligature falls off before the wound is filled up by granulations or, on the other hand, it only comes away afterwards.

In the first instance the blood continues to flow, while in the second the ligature can only be removed by an incision made in the regenerated tissues.

He describes fistula in ano with much care, and distinguishes two kinds, internal and external. The operation for fistula with an internal opening into the rectum, he describes as being accomplished by gradual constriction with a lead wire, or by section with the knife, guided by the finger in the rectum, or a canula of wood. This method has been little improved on since this time.

In his chapter on amputation of limbs, which in his day was avoided as much as possible on account of the fear of hemorrhage, he mentions the question of putting to sleep the unfortunate patient in order to avoid the pain of the incision, and I here give the exact Latin text of what he says regarding this:

Suntque (chirurgici) dant medicinas obdormitivas, ut patientes non sentiant incisionem, velut opium, succus morellae, hyosciami, mandragorae, cicutae, lactucae. Imbibunt in eis spongiam novam, et permittunt eam ad solam exsicarri; et quando erit necesse, mittunt illam spongiam in aqua calida, et dant eam ad odorandum tantum usquequo patientes capiant somnum. Et postea, cum alia spongia in aceto infusa, naribus apelicata expergefaciunt et evigilant eos.

This wonderful method of inhalation of anesthetics that the physicians of antiquity had already thought of, is consequently found distinctly expressed in the 13th century, but it took 600 years more for the realization of these dreams to be put into execution.

De Mondeville should surely occupy one of the highest places among the surgeons of the 13th Century, not only for the priority of many things, but from his talents and the high position that he occupied both socially and as a teacher. Educated in a healthy philosophical atmosphere, the mind enriched by the perusal of the best writers of antiquity, learned in medicine, familiar with both practical and theoretical surgery, surgeon to the Court of France, an enemy to quackery, a man of science, above all with an independent character, de Mondeville appears to us as one of the greatest glories of this epoch.

It is probable that de Mondeville was the first to demonstrate that the formation of pus was not necessary for the cicatrisation of wounds, and he thus may be said to have a priority of 600 years over the practice of Lister. Although there is a great space of time separating de Mondeville's method and the modern practice of asepsis and antisepsis, there was also a long lapse of time between his practice and those of the ancient surgeons. He was a pioneer in this direction, but other more complete and scientific works were necessary in order to prove in the minds of men the truth of his teachings.

871 BEACON STREET.

